# Participation in group-based physical activity programmes for adults in Germany and associated factors: data from a nationwide cohort study

**DOI:** 10.1186/s12889-018-6233-8

**Published:** 2018-12-12

**Authors:** Susanne Jordan, Susanne Krug, Elena von der Lippe

**Affiliations:** Robert Koch Institute, Department of Epidemiology and Health Monitoring, Box 65 0 61, 13302 Berlin, PO Germany

**Keywords:** Health behaviour change programme, Group programme, Health promotion, Prevention, Training course, Physical activity, Health-related behaviour, Cohort study, German health interview and examination survey for adults (DEGS1)

## Abstract

**Background:**

Characteristics of different participation groups can provide important information to increase participation in group-based physical activity programmes (GPAPs). This study examined four types of participation in GPAPs and the factors that characterised these participant groups.

**Methods:**

The present sample comprised 3219 participants. The analyses were based on data from the ‘German Health Interview and Examination Survey for Adults’ (t1) conducted in 2009–2011, which included 3959 people who had participated in the ‘German National Health Interview and Examination Survey 1998’ (t0). The outcome variable was participation in GPAPs, classified in four groups: ‘once at t1’ (participation only at t1), ‘twice’ (participation at t0 and t1), ‘once at t0’ (participation only at t0) and ‘no’ (no participation). Predictor variables were sex, age, educational level, income, sports activity, self-rated health and counselling for physical activity, measured at t0 and t1. Frequencies with 95% confidence intervals (CI) for each group were calculated. Four stepwise logistic regression models with estimated odds ratios (OR) were used to determine group differences.

**Results:**

The largest participant group was ‘no’ (80.8%). Among those who participated in GPAPs, the ‘once at t1’ group was the largest (13.1%), followed by the ‘once at t0’ (4.0%) and ‘twice’ (2.1%) groups. ‘Once at t1’ participation was associated with female sex (OR 2.58), being active in sports (OR 6.59), a high level of education (OR 1.88). If additionally health status and the physician’s counselling are included into the models, then having fair/poor/very poor health (OR 1.71) and having had physician counselling on physical activity (OR 2.50) are relevant factors. For ‘twice’ participation, being female (OR 5.19) and practising sports (OR 4.51) were predictors.

**Conclusions:**

GPAPs should be tailored to build on previous experience of sports activities and to reach men as well as people with low education, groups that have been the least reached. To reach more people and encourage participation in GPAPs, providing opportunity for physician counselling for physical activity may be promising, especially with groups of poorer health.

## Background

The promotion of physical activity is a priority for reducing lifestyle-related risk factors for noncommunicable diseases, which comprise the dominating burden of disease in high income countries [1, 2]. For Germany, promotion of physical activity is also important because only half of the population (or less, depending on the study referenced) meet the World Health Organization (WHO) recommendations for physical activity [3–5].

Various strategies are used to promote physical activity, including environmental and policy approaches, community-wide and mass media campaigns, and behavioural and social approaches [6, 7]. Different reviews and guidelines based on peer-reviewed studies show that specific interventions can have moderate effects on physical activity (self-reported and measured by cardio-respiratory fitness), although the relationships between intervention components and effects are not clear [7–9]. The WHO noted that in general, strategies that have multiple components and are adjusted to the local context are the most effective [10].

In Germany, health behaviour change programmes are widespread strategies to promote physical activity in adults [11]. These interventions use behavioural training methods, mainly through group-based courses focused on learning and maintaining physical activity practices. They differ from general sports programmes since they are based on a structured approach for health promotion. Our study refers to these programmes as group-based physical activity programmes (GPAPs). Different studies have reported GPAPs are promising for promoting physical activity at a population level. These programmes offer fitness instruction, often at low/no charge to participants, and provide social support through the group context [7]. Providers in Germany include sport clubs, adult education centres, companies, commercial providers (e.g. fitness studios) and statutory health insurance funds (which cover 86% of the population) [11, 12]. Programmes through health insurance funds also have a statutory order to contribute to reducing health inequality [13]. An evaluation of GPAPs showed they enhanced health behaviour patterns that later diminished in size, but remained higher than before programme participation [14]. These behaviour change effects are also found in comparable interventions from other countries [15].

Participation in GPAPs in Germany has increased about 6-fold in the last decade [16, 17], even considering the changed age structure [17]. However, it is necessary to increase investment in promoting GPAPs, given the low percentage of the population that has achieved physical activity levels necessary for positive health effects. To increase participation in GPAPs at a population level, it is essential to know more about the characteristics of participants and non-participants. In general, factors that influence participation in health behaviour change programmes (including GPAPs) at the population level in Germany are sex, age, education, social status, general health-related attitudes, social support, health behaviour and health status [17–21]. Therefore the probability of participation in health behaviour change programmes in Germany is increased when population groups are female, of older age, have a middle/high level of education or social status, live with a partner, are chronically ill or have a low level of self-assessed health status. Participation is also increased in those with pronounced health consciousness or who have healthy lifestyles (e.g. eating three or more portions vegetables and fruits per day, being physically active for 2.5 or more hours a week or being a non-smoker) [17–21].

Current findings about factors influencing participation in GPAPs in Germany are from cross-sectional studies. To date, there are no available data on proportions and factors associated with GPAP participation categories that could inform strategies to increase GPAP participation at the population level. Our study aimed to clarify the proportions of ‘once at t1’, ‘twice’, ‘once at t0’ and ‘no’ participation in GPAPs, and determine what distinguishes these groups with regard to demographic and socioeconomic factors, sports behaviour, health status and physician counselling on physical activity. The results will provide data to allow better tailoring of GPAPs for adults to increase GPAPs participation.

## Methods

### Study design

The analyses were based on data from the ‘German Health Interview and Examination Survey for Adults’ (DEGS1). That survey included 3959 persons who had previously participated in the ‘German National Health Interview and Examination Survey 1998’ (GNHIES98) (response rate 62%) [22]. All 6402 GNHIES98 participants were re-invited to join the DEGS1 cohort, and 37% chose to participate in the DEGS1. Baseline data collection for the GNHIES98 (t0) occurred from October 1997 to March 1999 [23], and for the DEGS1 (t1) from November 2008 to December 2011 [22]. Participants in both surveys were residents of Germany aged 18–79 years. Both surveys were based on two-stage stratified random sampling from local population registers. Data were collected using standardised computer-assisted personal interviews, standardised measurements/tests and a self-administered questionnaire covering physical, mental and social aspects of health. The detailed concept and design of the GNHIES98 and DEGS1 are described elsewhere [22, 24–26].

The DEGS1 was approved by the Federal and State Commissioners for Data Protection and the Charité-Universitätsmedizin Berlin Ethics Committee (No. EA2/047/08). All participants provided written informed consent. The GNHIES98 was approved by the Board of the Federal Commissioner for Data Protection Berlin.

### Sample description and participants

Our analysis included data from adults who participated in both survey waves and were aged 30–79 years at t1 (18 years and older at baseline/t0). This ensured that participants had opportunity for twice participation in GPAPs at both surveys, as the surveys only included people aged up to 79 years. Furthermore, the analysis was limited to respondents insured under statutory health insurance at follow-up. Statutory health insurance represents more than 86% of the German population and is a major provider of GPAPs. Participants were asked what type of health insurance they had, and a distinction was made between private and statutory health insurance. Our sample comprised 3219 participants.

### Measures

#### Outcome variable

##### Programme participation

In both survey waves, standardised self-administered questionnaires were used to obtain information on GPAPs participation. After a brief introduction, participants were asked whether they had participated in a GPAP during the last 12 months, and if yes, the activity in which they participated [17, 19]. Both surveys used the same questions with minor variations in wording. At t0 (GNHIES98) the question was: ‘There are a number of health promotion programmes organised by health insurance funds, adult education centres, health authorities, private providers or self-help groups focusing on topics like for example diet, physical activity, relaxation and sport or fitness. Have you ever taken part in such measures (courses, exercises, consultations)? Multiple answers allowed’. Various health behaviours were listed: weight reduction, healthy diet, back or spinal gymnastics (back training), relaxation/stress reduction, cessation of smoking, cessation of alcohol abuse, cessation of drug use, other. Response options for each: ‘no’, ‘yes, in the last 12 months’ or ‘yes, more than 12 months ago’ (only the first two categories were included in our analyses). [In German: ‚Es gibt eine Reihe von Maßnahmen zur Gesundheitsförderung, die z.B. von Krankenkassen, Volkshochschulen, Gesundheitsämtern, privaten Anbietern oder Selbsthilfegruppen durchgeführt werden und sich beispielsweise mit Ernährung, Bewegung, Entspannung und Sport oder Fitness befassen. Haben Sie an solchen Maßnahmen (Kurse, Übungen, Beratungen) schon einmal teilgenommen? Sie können mehreres ankreuzen‘: Es wurden verschiedene Gesundheitsverhalten aufgelistet: Gewichtsreduktion, gesunde Ernährung, Rücken- oder Wirbelsäulengymnastik (Rückenschule), Entspannung/Stressbewältigung, Raucherentwöhnung, Alkoholentwöhnung, Drogenentwöhnung, sonstige. Jeweilige Antwortoptionen: ‚nein‘, ‚ja, in den vergangenen 12 Monaten‘ oder ‚ja, vor mehr als 12 Monaten‘ (nur die ersten beiden Kategorien gingen in unsere Analysen ein)]. At t1 (DEGS1) the question was: ‘There are a number of health promotion programmes organised by various providers and focusing on topics like for example diet, physical activity, relaxation and sport or fitness. Some of these programmes are financed by health insurance funds. Have you taken part in programmes of this kind (courses, exercises, consultations) in the last 12 months? If yes, please indicate which programmes you have attended in the last 12 months and how they have been funded. Multiple answers allowed.’ Various health behaviours were listed: weight reduction, healthy diet, gymnastics, relaxation, fitness/recreational sports, relaxation/stress reduction, cessation of smoking, cessation of alcohol abuse, cessation of medication misuse, other. Each could be ticked with: ‘yes, in the last 12 months’ and how these were financed (the latter was not included in our analyses)]. [In German: ‚Es gibt eine Reihe von Maßnahmen zur Gesundheitsförderung, die von verschiedenen Anbietern durchgeführt werden und die sich beispielsweise mit Ernährung, Bewegung, Entspannung und Sport oder Fitness befassen. Teilweise werden solche Maßnahmen von den Krankenversicherungen finanziert. Haben Sie an solchen Maßnahmen (Kurse, Übungen, Beratungen) in den letzten 12 Monaten teilgenommen? ‚Wenn ja, bitte geben Sie an, welche Maßnahmen Sie in den letzten 12 Monaten besucht haben und wie diese finanziert waren. Mehrfachantworten möglich‘. Es wurden verschiedene Gesundheitsverhalten aufgelistet: Gewichtsreduktion, gesunde Ernährung, Gymnastik, Entspannung/Stressbewältigung, Fitness/Ausgleichssport, Raucherentwöhnung, Alkoholentwöhnung, Medikamentenentwöhnung, sonstige. Es konnte jeweils angekreuzt werden: ‚ja, in den vergangenen 12 Monaten‘ und wie diese finanziert wurden (letzteres ging nicht in unsere Analysen ein)]. The GNHIES98 response options ‘back or spinal gymnastics (back training)’ were adapted in the DEGS1 to the current physical activity programme names (‘gymnastics’ and ‘fitness/recreational sport’), which were based on the *Guideline Prevention* published by the National Association of Statutory Health Insurance Funds [13]. These two options were combined into a ‘physical activity’ variable. For statistical analyses of GPAP participation, four categories were defined based on GPAP participation/non-participation in both surveys: ‘twice’ (participation at t0 and t1), ‘once at t1’ (no participation at t0, but participation at t1), ‘once at t0’ (participation at t0, but not at t1) and ‘no’ (no participation at t0 or t1).

#### Predictor variables

The selection of variables included in the analyses was therefore based on the results of existing research on factors influencing participation in health behaviour change programmes, with a focus on research in Germany [17, 20, 27]. A number of relevant factors were identified: demographic characteristics (sex and age), socioeconomic characteristics (educational level), sports behaviour (sports activity), health status (self-assessed) and health system-related enabling factors (e.g. physical activity counselling by a physician). Income was included because GPAPs in Germany are not free of charge. Depending on the provider, programmes require a participation fee, which can be partly reimbursed for courses offered by a statutory health insurance fund [13].

##### Demographic characteristics

Participants’ sex and age were included in the analyses. Age was categorised into four groups: 18–29 years, 30–44 years, 45–64 years and 65–79 years. Respondents younger than 30 years at t1 were not included in the analysis, and are therefore not shown in the tables. This was because they did not have an opportunity to participate in GPAP twice, because the age group under study in GNHIES98 (t0) was 18–79 years.

##### Socioeconomic characteristics

Educational level was calculated according to the International Standard Classification of Education, based on participants’ school and vocational education grouped into three levels: low, middle and high education [28, 29]. The need-weighted household net income (net equivalent income) was used as an indicator for income, calculated from household estimated monthly net income and number of individuals living in that household [30]. Missing values for household net income were imputed using a regression model [30, 31]. Based on the general convention on income poverty risk, defined as an income of less than 60% of the needs-weighted median income, three categories were created: below 60, 60–150% and above 150%.

##### Sports behaviour

Sports activity is the health behaviour associated with participation in GPAPs. To assess the frequency of current sports activity, participants were asked how often they practiced sports per week. The last 3 months were given as the reference period. Response categories were: ‘no sports activities’, ‘less than 1 hour per week’, ‘regularly 1–2 hours per week’, ‘regularly 2–4 hours per week’ and ‘regularly more than 4 hours per week’.

##### Health status

Health status was collected by a question on self-rated health that is widely used in surveys and recommended by the WHO [32]. Participants were asked ‘How is your health in general?’ The five response options were combined into two groups: ‘very good/good’ and ‘fair/poor/very poor’.

##### Counselling on physical activity

A health system-related enabling factor for participating in health behaviour change programmes was physical activity counselling/advice from physicians, which appears to be effective in increasing individual physical activity [33–35]. The questionnaire item (self-reported) asked if participants had received physical activity counselling from a primary healthcare physician over the 12 months before the interview. As this question was only asked of persons up to age 64 years, the variable was added in the regression models last, and results are only available for persons up to this age.

#### Statistical analyses

Analyses were conducted with a weighting factor to: account for the clustered sampling design; adjust for the distribution of the sample by sex, age, educational status, federal state and type of municipality (to match the German population at t0 and t1); and adjust for participants’ response behaviour [22]. Statistical analyses were performed using survey procedures for complex samples in IBM SPSS v20. Frequencies and 95% confidence intervals (CI) for the participation groups were calculated. Differences were regarded as statistically significant if the *p*-value (taking into account the weighting and survey design) was less than 0.05. Given the small number of cases in some subgroups, the analyses could not be stratified by sex. A ‘lasagne plot’ was used to visualise the types of participation in GPAPs at t0 and t1 [22, 36].

Logistic regression models with estimated odds ratios (OR) were used to calculate factors associated with participation groups and determine group differences (reference group ‘once at t0’ for the ‘twice’ group and reference group ‘no’ for the group ‘once at t1’). The OR indicated the association between participation in GPAP and a predictor. For example, in the ‘once at t1’ group, an OR of 2.00 for women (reference group men) indicated that the odds for women’s GPAP participation at t1 were twice as high as for men compared to women and men who had not participated in GPAP at t0 and t1.

Variables were added to the model in a stepwise fashion to identify factors relevant for each group. Data from both t0 and t1 were included in the models to show their individual explanatory contributions at the two time points. Model 1 was adjusted for demographic and socioeconomic characteristics at t0 and t1 (sex, age, education, income) (but sex and education at t1 were not included, because they are assumed to be consistent for persons over 30 years). Model 2 comprised Model 1 and sports behaviour at t0 and t1. Model 3 included Model 2 plus health status at t0 and t1 and Model 4 contained Model 3 and physician physical activity counselling at t0 and t1. Statistically significant results (*p* < 0.05) are reported in the Results section.

## Results

### Sample characteristics

Table 1 shows the characteristics of the survey population at t0 and t1 differentiated by demographic (sex and age) and socioeconomic characteristics (education and income). Table 1 also shows the distribution of variables used in further analyses: programme participation, sports activity, self-rated health and counselling for physical activity by a physician. Because of missing values in at least one independent variable, only 3178 respondents could be included in the multivariate analyses.

**Table 1:** Sample description (*N* = 3219)

	n (t0)	n (t1)	Total (t1)	Missing (t1)
	% (weighted)	% (weighted)	N (unweighted)	% (unweighted)
Demographic characteristics				
*Sex*				0.0
Women	52.8	52.8	1788	
Men	47.2	47.2	1431	
*Age*				0.0
18–29 years	22.4^a^	–	–	
30–44 years	38.6	30.6	719	
45–64 years	35.8	44.4	1542	
65–79 years	3.2	25.0	958	
Socioeconomic characteristics				
*Educational level*				0.3
Low	20.5	17.5	366	
Middle	60.4	55.8	1844	
High	19.2	26.7	1000	
*Income*				0.0
Below 60%	18.4	14.4	435	
60–150%	64.9	65.8	2164	
Above 150%	16.7	19.8	620	
Programme participation				
*Participation in a group-based physical activity programme in the last 12 months*				1.3
Yes	6.1	15.1	573	
No	93.9	84.9	2605	
Sports behaviour				
*Sports activity (in the last 3 months)*				1.0
No sports	46.4	38.9	1140	
Less than 1 h per week	16.1	15.0	517	
Regularly 1–2 h per week	17.4	26.2	863	
Regularly 2–4 h per week	12.4	13.3	447	
Regularly more than 4 h per week	7.7	6.6	219	
*Sports activity aggregated (in the last 3 months)*				1.0
No sports	46.4	38.9	1140	
Sports activity per week	53.6	61.1	2046	
Health status				
*Subjective health status*				0.5
Good/very good	69.3	68.4	2211	
Fair/poor/very poor	30.7	31.6	991	
Enabling factor counselling				
*Physician physical activity counselling*^b^				30.5
No	91.1	90.6	2022	
Yes	8.9	9.4	214	

^a^Including 10 cases with the age of 17

^b^Only persons up to age 64 years

### Descriptive analyses

**Fig. 1:**
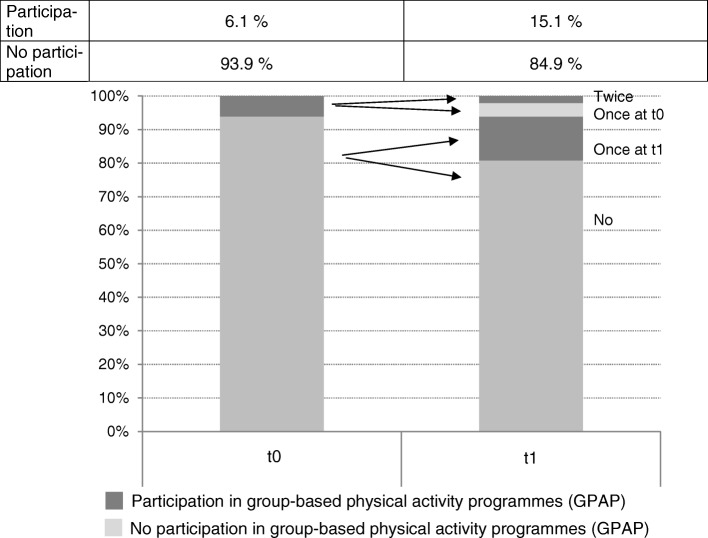
Plot and distribution table of different participation groups in GPAP in adults at t0 and t1

GPAP participation increased from 6.1% (t0) to 15.1% (t1). Figure 1 illustrates the different participation groups in GPAPs at t0 and t1. The largest group was non-participants, with no participation at t0 or t1 (‘no’ 80.8, 95% CI 79.2–82.4). The largest group among GPAPs participants was ‘once at t1’ participants, who started GPAP at t1 (13.1, 95% CI 11.7–14.5), followed by the ‘once at t0’ group with participation at t0 but not at t1 (4.0, 95% CI 3.4–4.9). The ‘twice’ group, who participated at both measurement points, was the smallest participant group at 2.1% (95% CI 1.7–2.6).

Table 2 presents the characteristics of the four groups at t1 by demographic and socioeconomic factors, health status, sports behaviour and physical activity counselling by a physician. Most of the potential predictor variables showed significant differences with the outcome variable for one or more group comparisons. What was noticeable was the difference between the three groups that had participated in GPAP and the ‘no’ group in terms of sports behaviour. The proportion who reported sports behaviour in the last 3 months at the two times was between 73.2 and 91.4%, whereas the proportion for the group without GPAP experience was only 55.3%.

**Table 2:** Characteristics of different participation groups in group-based physical activity programmes at t1

Participation	‘Once at t1’ *n* = 479 % (95% CI)	‘Twice’ *n* = 94 % (95% CI)	‘Once at t0’ *n* = 149 % (95% CI)	‘No’ *n* = 2456 % (95% CI)	Total *n* = 3178 % (95% CI)	*p*-value
Total	13.1 (11.7–14.5)	2.1 (1.7–2.6)	4.0 (3.4–4.9)	80.8 (79.2–82.4)		
Demographic characteristics						
*Sex*						
Women	68.3 (63.4–72.9)	79.9 (67.9–88.3)	62.8 (53.9–70.8)	48.8 (46.4–51.1)	52.5 (50.5–54.6)	
Men	31.7 (27.1–36.6)	20.1 (11.7–32.1)	37.2 (29.2–46.1)	51.2 (48.9–53.6)	47.5 (45.4–49.5)	0.000
*Age*						
30–44 years	31.6 (26.3–37.4)	10.7 (5.7–19.4)	24.2 (17.2–32.9)	31.6 (28.6–34.7)	30.9 (28.1–33.7)	
45–64 years	43.0 (37.8–48.4)	52.3 (41.2–63.2)	49.6 (41.6–57.6)	44.4 (41.4–47.4)	44.6 (42.0–47.2)	
65–79 years	25.4 (21.3–30.0)	37.0 (27.0–48.1)	26.2 (19.5–34.2)	24.1 (22.1–26.2)	24.6 (22.7–26.6)	0.011
Socioeconomic characteristics						
*Educational level*						
Low	9.7 (6.6–14.1)	16.1 (9.0–27.2)	15.4 (9.5–24.2)	18.6 (16.0–21.5)	17.3 (15.0–19.7)	
Middle	54.9 (49.5–60.2)	48.7 (38.4–59.2)	58.4 (49.7–66.5)	56.1 (53.2–59.0)	55.9 (53.3–58.4)	
High	35.4 (30.3–40.7)	35.2 (25.6–46.1)	26.2 (18.9–35.1)	25.3 (23.0–27.7)	26.8 (24.7–29.1)	0.000
*Income*						
Below 60%	9.9 (7.0–13.9)	11.7 (6.3–20.5)	9.9 (5.4–17.2)	15.4 (13.3–17.7)	14.4 (12.5–16.4)	
60–150%	66.5 (60.4–72.1)	68.1 (57.7–76.9)	72.2 (63.2–79.8)	65.3 (62.9–67.6)	65.8 (63.6–68.0)	
Above 150%	23.6 (18.7–29.3)	20.3 (13.1–30.0)	17.9 (11.9–26.0)	19.3 (17.1–21.7)	19.8 (17.8–22.0)	0.052
Sports behaviour						
*Sports activity aggregated (in the last 3 months)*						
No sports	10.8 (8.1–14.5)	8.6 (4.6–15.5)	26.8 (18.8–36.7)	44.7 (42.0–47.4)	38.7 (36.4–41.1)	
Sports activity per week	89.2 (85.5–91.9)	91.4 (84.5–95.4)	73.2 (63.3–81.2)	55.3 (52.6–58.0)	61.3 (58.9–63.6)	0.000
Health status						
*Subjective health status*						
Good/very good	66.5 (60.9–71.6)	66.8 (57.1–75.3)	65.0 (55.9–73.1)	69.3 (66.6–71.8)	68.7 (66.3–70.9)	
Fair/poor/very poor	33.5 (28.4–39.1)	33.2 (24.7–42.9)	35.0 (26.9–44.1)	30.7 (28.2–33.4)	31.3 (29.1–33.7)	0.515
Enabling factor						
*Physician physical activity counselling*						
No	80.5 (75.4–84.8)	84.3 (72.2–91.7)	91.7 (85.0–95.6)	92.3 (90.7–93.7)	90.6 (89.1–92.0)	
Yes	19.5 (15.2–24.6)	15.7 (8.3–27.8)	8.3 (4.4–15.0)	7.7 (6.3–9.3)	9.4 (8.0–10.9)	0.000

CI, confidence interval

### Regression analysis

The stepwise multivariate logistic regression showed significant associations between predictor and outcome variables for the ‘twice’ group (reference ‘once at t0’ group) and ‘once at t1’ group (reference: ‘no’). The results are presented in Tables 3 and 4 for each group and model.

**Table 3:** Factors for ‘once at t1’ participation in group-based physical activity programmes

	Model 1 Odds ratio (95% CI)	Model 2 Odds ratio (95% CI)	Model 3 Odds ratio (95% CI)	Model 4 Odds ratio (95% CI)
Demographic characteristics				
*Sex t0*				
Women	2.55*** (1.98–3.29)	2.54*** (1.95–3.32)	2.58*** (1.98–3.38)	2.69*** (1.90–3.80)
Men	Ref.	Ref.	Ref.	Ref.
*Age t0*				
18–29 years	0.99 (0.57–1.72)	0.92 (0.52–1.61)	0.99 (0.55–1.78)	0.96 (0.53–1.74)
30–44 years	Ref.	Ref.	Ref.	Ref.
45–64 years	1.02 (0.68–1.54)	1.08 (0.71–1.64)	1.06 (0.69–1.61)	1.15 (0.74–1.81)
65–79 years	0.40 (0.15–1.08)	0.52 (0.18–1.50)	0.46 (0.16–1.35)	–
*Age t1*				
30–44 years	Ref.	Ref.	Ref.	Ref.
45–64 years	0.92 (0.57–1.48)	0.85 (0.52–1.39)	0.83 (0.49–1.40)	0.77 (0.46–1.30)
65–79 years	1.18 (0.61–2.28)	1.15 (0.58–2.26)	1.13 (0.56–2.31)	–
Socioeconomic characteristics				
*Educational level t0*				
Low	Ref.	Ref.	Ref.	Ref.
Middle	1.82** (1.18–2.80)	1.41 (0.92–2.16)	1.47 (0.96–2.26)	1.19 (0.70–2.04)
High	2.60*** (1.61–4.20)	1.80* (1.12–2.91)	1.88* (1.16–3.04)	1.59 (0.85–2.97)
*Income t0*				
Below 60%	Ref.	Ref.	Ref.	Ref.
60–150%	1.12 (0.72–1.72)	1.12 (0.73–1.73)	1.12 (0.74–1.70)	1.19 (0.71–1.98)
Above 150%	1.34 (0.84–2.15)	1.26 (0.80–1.98)	1.27 (0.82–1.99)	1.01 (0.55–1.85)
*Income t1*				
Below 60%	Ref.	Ref.	Ref.	Ref.
60–150%	1.44 (0.94–2.21)	1.27 (0.82–1.98)	1.37 (0.89–2.10)	1.22 (0.73–2.04)
Above 150%	1.52 (0.90–2.57)	1.17 (0.69–1.98)	1.30 (0.78–2.17)	1.21 (0.66–2.22)
Sports behaviour				
*Sports activity aggregated (in the last 3 months) t0*				
No sports		Ref.	Ref.	Ref.
Sports activity per week		1.00 (0.78–1.28)	1.04 (0.82–1.33)	1.08 (0.78–1.50)
*Sports activity aggregated (in the last 3 months) t1*				
No sports		Ref.	Ref.	Ref.
Sports activity per week		6.20*** (4.20–9.14)	6.59*** (4.44–9.80)	6.46*** (4.14–10.10)
Health status				
*Subjective health status t0*				
Good/very good			Ref.	Ref.
Fair/poor/very poor			0.78 (0.54–1.13)	0.82 (0.52–1.28)
*Subjective health status t1*				
Good/very good			Ref.	Ref.
Fair/poor/very poor			1.94*** (1.42–2.66)	1.71** (1.14–2.57)
Enabling factor				
*Physician physical activity counselling t0*				
No				Ref.
Yes				0.99 (0.64–1.51)
*Physician physical activity counselling t1*				
No				Ref.
Yes				2.50*** (1.64–3.80)
Nagelkerke	0.065	0.164	0.175	0.177

Results from logistic regression analyses for ‘once at t1’ participation in group-based physical activity programmes, reference group ‘no’ participation in group-based physical activity programmes). CI, confidence interval. Ref., reference group. Significant associations are shown in bold and as *p < 0.05, **p < 0.01, and ***p < 0.001. Model 1 included sex, age, education and income at t0 and age and income at t1 (sex and education were assumed to be constant for persons over 30 years); Model 2 included Model 1 variables plus sports activity at t0 and t1; Model 3 included Model 2 variables plus subjective health status at t0 and t1; Model 4 included Model 3 variables plus physician physical activity counselling at t0 and t1

**Table 4:** Factors for ‘twice’ participation in group-based physical activity programmes

	Model 1 Odds ratio (95% CI)	Model 2 Odds ratio (95% CI)	Model 3 Odds ratio (95% CI)	Model 4 Odds ratio (95% CI)
Demographic characteristics				
*Sex t0*				
Women	3.00** (1.45–6.21)	2.88** (1.35–6.13)	3.05** (1.42–6.57)	5.19** (1.69–15.96)
Men	Ref.	Ref.	Ref.	Ref.
*Age t0*				
17–29 years	0.91 (0.16–5.12)	0.92 (0.16–5.22)	1.04 (0.19–5.79)	1.18 (0.20–7.13)
30–44 years	Ref.	Ref.	Ref.	Ref.
45–64 years	2.59* (1.03–6.51)	2.36 (0.94–5.89)	2.29 (0.88–5.92)	2.61 (0.87–7.84)
65–79 years	2.57 (0.18–37.22)	1.59 (0.11–22.68)	1.47 (0.10–20.98)	–
*Age t1*				
30–44 years	Ref.	Ref.	Ref	Ref
45–64 years	1.38 (0.31–6.12)	1.43 (0.31–6.52)	1.58 (0.35–7.14)	1.69 (0.35–8.12)
65–79 years	1.06 (0.17–6.50)	1.33 (0.22–7.94)	1.57 (0.26–9.60)	–
Socioeconomic characteristics				
*Educational level t0*				
Low	Ref.	Ref.	Ref.	Ref.
Middle	1.21 (0.50–2.93)	1.37 (0.54–3.44)	1.46 (0.61–3.54)	2.75 (0.72–10.61)
High	2.50 (0.95–6.61)	2.56 (0.90–7.31)	2.87* (1.02–8.13)	4.17 (0.91–19.16)
*Income t0*				
Below 60%	Ref.	Ref.	Ref.	Ref.
60–150%	0.73 (0.28–1.90)	0.54 (0.22–1.35)	0.56 (0.22–1.38)	0.28 (0.08–1.00)
Above 150%	0.82 (0.26–2.59)	0.68 (0.21–2.18)	0.65 (0.20–2.08)	0.39 (0.08–1.97)
*Income t1*				
Below 60%	Ref.	Ref.	Ref.	Ref.
60–150%	0.72 (0.26–1.99)	0.80 (0.28–2.30)	0.69 (0.24–1.98)	0.69 (0.17–2.74)
Above 150%	0.79 (0.26–2.41)	0.89 (0.29–2.77)	0.79 (0.24–2.60)	1.3 (0.30–5.73)
Sports behaviour				
*Sports activity aggregated (in the last 3 months) t0*				
No sports		Ref.	Ref.	Ref.
Sports activity per week	–	1.50 (0.67–3.35)	1.44 (0.64–3.25)	1.73 (0.48–6.23)
*Sports activity aggregated (in the last 3 months) t1*				
No sports		Ref.	Ref	Ref.
Sports activity per week		3.71** (1.50–9.17)	3.86** (1.51–9.82)	4.51* (1.34–15.24)
Health status				
*Subjective health status t0*				
Good/very good			Ref.	Ref.
Fair/poor/very poor			0.78 (0.40–1.52)	0.54 (0.19–1.56)
*Subjective health status t1*				
Good/very good			Ref.	Ref.
Fair/poor/very poor			1.21 (0.61–2.41)	1.71 (0.56–5.23)
Enabling factor				
*Physician physical activity counselling t0*				
No				Ref.
Yes				1.36 (0.49–3.77)
*Physician physical activity counselling t1*				
No				Ref.
Yes				3.15 (0.98–10.09)
Nagelkerke	0.137	0.196	0.202	0.288

Results from logistic regression analyses for ‘twice’ participation in group-based physical activity programmes (reference group ‘once at t0’ participated in group-based physical activity programmes). CI, confidence interval. Ref., reference group. Significant associations are shown in bold and as *p < 0.05, **p < 0.01, and ***p < 0.001. Model 1 included sex, age, education and income at t0 and age and income at t1 (sex and education were assumed to be constant for persons over 30 years); Model 2 included Model 1 variables plus sports activity at t0 and t1; Model 3 included Model 2 variables plus subjective health status at t0 and t1; Model 4 included Model 3 variables plus physician physical activity counselling at t0 and t1

For ‘once at t1’ participants, women had a greater chance of GPAPs participation (Table 3). Depending on the regression model, the OR was about 2.60 (e.g. Model 4: OR 2.69, 95% CI 1.90–3.80). Sports behaviour showed the largest effect with an OR above 6 (e.g. Model 4: OR 6.46, 95% CI 4.14–10.10). Compared with low education at t0, a medium or high education level facilitated ‘once at t1’ participation by 1.80 (95% CI 1.12–2.91) to 2.60 (95% CI 1.61–4.20), depending on the model and whether the medium or high education group was being examined. In Model 4 educational level showed no significant result anymore. Fair/poor/very poor health status and having had physical activity counselling were associated with smaller chances of being an ‘once at t1’ participant (e.g. Model 4: OR 1.71, 95% CI 1.14–2.57 and OR 2.50, 95% CI 1.64–3.80, respectively). No significant results were found for age and income and all of the factors at t0 with the exception of the educational level (see above).

Being female was the strongest predictor for ‘twice’ participation, with ORs between 2.88 (95% CI 1.35–6.13) and 5.19 (95% CI 1.69–15.96), depending on the model. Income, health status and physical activity counselling had no influence. Age and educational level showed only one association in Model 1 or 3. No further significant results were found for the factors at t0. However, practising sports activity showed an OR between 3.71 (95% CI 1.50–9.17) and 4.51 (95% CI 1.34–15.24) compared with not practising sports. The results for ‘twice’ participation are shown in Table 4.

## Discussion

This study aimed to clarify the proportion and factors characterising ‘once at t1’, ‘twice’, ‘once at t0’ and ‘no’ participation in GPAPs using nationwide cohort data from Germany. Regarding participation proportions for the four groups, this study showed that participation in GPAPs increased from about one in 16 people in 1998 to one in seven people in 2009–2011. However, 80.8% did not participate in a GPAP in either of the waves, meaning the ‘no’ group was the largest. This is consistent with expectations, because generally only a certain proportion of the population can be reached with these types of programmes. Among those that had experienced a GPAP, the ‘once at t1’ participation group (that started a GPAP at the second wave) was the largest; at 13.1% of participants, it was 6-fold larger than the ‘twice’ group (2.1%) and 3-fold larger than the ‘once at t0’ group (4.0%). This increase in participation is consistent with parallel developments; for example, fitness studio membership in Germany increased in a comparable period from 4.39 million in 2003 to 6.54 million in 2009 [37]. In addition, sports clubs and adult education centres are increasingly offering health-oriented group courses on physical activity, and have experienced increasing demand and members [38]. This corresponds with the observation that one-third of the adult population (as at 2009) reported they paid attention to adequate physical activity [4]. Our study is the first to show participation rates in GPAP at a population level, based on data that considered previous experience of GPAP participation at an individual level. We also showed that despite the overall increase, the increase in participation rates did not mean that those who participated at t0 participated 10 years later (t1). However, this difference might be explained by the long time interval between the two waves in our study. We do not know whether people in these groups had visited a GPAP in this period.

We observed common characteristics and differences in factors characterising the four participation groups. ‘Once at t1’ participation was associated with being female, practising sports in the last 3 months, having medium or high level of education, being in fair/poor/very poor health and having had counselling on physical activity from a physician in the last 12 months. For ‘twice’ participation, female sex and sports activity in the last 3 months were the relevant factors with significant differences to the reference group. The ‘once at t0’ group was characterised by female sex and practising sports in the last 3 months. The ‘no’ group had the highest proportion of sports inactivity compared with the other groups and had an above-average proportion of men. In general, men participate less in prevention programmes [16, 17, 39] but practice more sports [4, 40]. Overall, it was noticeable that female sex and sporting behaviour increased the chance of GPAP participation. However, neither age nor income showed relevant differences in terms of participation in GPAPs. Health status and physician counselling on physical activity were only relevant for the ‘once at t1’ group. Almost none of the factors under study at t0 were associated with GPAPs participation, age at t0 in Model 1 in ‘twice’ participation was considered subordinate for the overall interpretation. This was unexpected, particularly with regard to participation in sport at t0, as other studies showed that previous participation in sport favours the retention/reactivation of sport or physical activity [41–43]. Our results showed that participation in prevention programmes was, at least in part, influenced by factors other than self-initiated sporting activity.

To our knowledge, the present study of GPAPs participation at the population level is the first of its kind. The results cannot be simply compared with findings from intervention studies focused on promoting physical activity where the influencing factors are known and specifically controlled (e.g. training material, participants of group training, trainer’s qualification, objective measurements of participants’ attitudes and behaviours). GPAPs participation examined in this study was based on ‘realist’ or everyday conditions as a type of health services research [15, 44], meaning that only self-report for GPAP participation and self-rated information on the studied factors were available. The advantage was that the data realistically reflect the use of the programmes at the population level. However, no data on GPAPs implementation conditions, recruitment, course composition and other details were available, which is often the case with research on health service use. Consequently, for discussion, it was only possible to draw on findings from intervention studies that differed from GPAPs in the present study. Intervention studies are either one-time measures to promote physical activity or research on other health behavioural changes under less realistic conditions. Therefore, these studies did not investigate twice (group) activity participation in two different time points with a long time period, and only analysed motives or mediators for starting participation in a physical activity programme. Therefore, we have summarised factors relevant for each participant group and discussed the relevance and interpretation of each factor.

### Demographic characteristics

Although the descriptive analysis (Table 2) shows a significant influence of age, age played no role in GPAPs participation. This contrasts with other studies [45, 46], but might be explained by other factors in the regression analyses having a stronger influence on participation. Female sex increased the chance of participation compared with the reference group. Consistent with other studies, our results show that males were generally underrepresented [47].

### Socioeconomic characteristics

Different income levels showed no significant differences for participation in GPAPs, consistent with other studies on health behaviour change programmes [20]. In contrast, educational level was associated with once at t1 participation, with middle and high education levels increasing the chance of starting participation in GPAPs. In general, these courses are dominated by those with middle and high educational levels [20, 48, 49].

### Sports behaviour (sports activity in the last 3 months)

Our study confirmed the observation that behavioural intervention programmes may reach those who need them the least [45, 50]. For example, people who twice participated in a web-based health promotion programme had healthier lifestyles than people who participated only once [45]. One study found that in repeaters, the motives for sporting activity were more pronounced [51]. In our study, sports activity in the last 3 months was relevant for all participant groups, especially the ‘once at t1’ and ‘twice’ groups, where sports activity was a strong predictor for participation in GPAPs. ‘Once at t0’ participation might be attributable to several reasons. The end of a sports programme (GPAP) is a critical point (decision-making moment) and often means the end of that activity. Other studies have shown that people who dropped out of a sports programme often found the programme, the trainer, the location or their current living situation less appropriate that those who continued participation [51]. Sports activity might indicate that starting another sports activity (e.g. a fitness studio) was a reason for ‘once at t0’ group [51, 52]. Some GPAPs explicitly state they serve to stimulate physical activity in general (for example courses offered by the statutory health insurance funds) [13]. In general, sport participation increased the chance of participating in GPAPs, meaning GPAPs reach people who are already active. Therefore, there is a risk that GPAPs will increase health inequalities (prevention dilemma). However, they are also able to motivate people who have been physically active as a result of physicians’ counselling.

### Health status

Health status was only relevant for the ‘once at t1’ group. Contrary to other studies, a worse state of health was not accompanied by less physical activity among ‘once at t0’ participants [43, 53]. People with fair/poor/very poor health had a greater chance of starting a GPAP than those with good/very good health. An individual perception of a poorer state of health may motivate people to improve their health behaviour, for example increasing physical activity. This is consistent with other studies that investigated how people with chronic illnesses can be reached to increase physical activity to reduce the risk of secondary illnesses (e.g. diabetes type 2 or cardiovascular disease) [54]. A GPAP seems a helpful motivator for starting physical activity, especially if a doctor has advised such activity [55].

### Counselling on physical activity

Physician counselling on physical activity was a health system-related enabling factor that was only relevant for the ‘once at t1’ group. In general, counselling or advice for brief physical activity (5–20 min) through primary care aims to raise consciousness of the relevance of physical activity and encourage people to increase/maintain their activity level [56]. Consistent with other studies [33–35, 57], we found physical activity counselling was effective in motivating and supporting people to start physical activity [56]. This may also be a cost-effective intervention that to improve physical activity, even at 12 months following the intervention [33]. However, there may be a risk that physician counselling may lead to widening health inequalities if people from vulnerable groups do not use primary care services [58].

#### Strength and limitations

A strength of this study was that it is the first to examine the population groups and factors that affect participation (‘once at t1’, ‘twice’, ‘once at t0’ and ‘no’) in GPAPs at two waves over a long time span. However, a weakness of our study design was that there was almost no information about the health status, sports behaviour or preventive activities between the two waves, and no information about the circumstances of the GPAPs visited by participants. It should also be noted that our study only included a limited number of factors, namely those that were expected to be directly related to participation in GPAPs. Further research on factors related to twice or one-time participation in GPAPs should integrate additional factors into the analyses, such as body weight (overweight and obesity), smoking habits, fruit and vegetable consumption [45, 55] or health-related quality of life, self-efficacy and enjoyment [48], all of which influenced participation in health behaviour change programmes in other studies.

Regarding the GPAP question, the response options at t0 and t1 are not fully identical, so that the comparability is not perfect and might have led to a small bias (with an underestimation at t0 of participants). In contrast to t1, the option fitness courses was not offered as an answer due to the fact that at this time these were not offered as health promotion programmes. However, there is no indication that a possible underreporting bias at t0 systematically skews the result with regard to the analysed influencing factors. The partly different wording of answer options was adapted to typical names and contents of GPAP during each measurement. This should make it easier for respondents to identify the programmes in which they actually took part in.

With regard to the association between GPAP and sporting activity, it should be noted that it cannot be completely ruled out that respondents might have considered GPAP participation as sporting activity. However, cross tabulation between sport activity and GPAP participation for GNHIES98 and DEGS1 showed that there were enough cases of respondents in the GPAP, who participate in GPAP and did not practice sport. Another aspect is that participation in GPAP refers to the last 12 months and sports activity refers to the last three months. We assume, thus, that the overlapping (people who said they did sport did so because of participation in GPAP) is relative small.

A limitation of the observed factors affects our conclusions based on the analyses of Model 4. With the inclusion of the enabling factor (physician physical activity counselling), only data from persons up to age 64 years could be inserted into the regression model because this information was only obtained from people aged up to 64 years. However, as counselling by a physician was identified as important in other studies, we integrated this variable into the model. Additionally, it must be considered that there could be a relation between the worse health status and a contact with a physician which would lead to a counselling. Unfortunately, with the current analysis we are not able to control for this.

## Conclusions

Various studies have shown that GPAP participation in Germany has increased over the last decade. However, our study is the first to provide insights about GPAP participation considering previous individual-level GPAP experience. Based on data from these two separate time points, this study specifies the proportions of people who participated once or twice in such programmes, those who are not reached, and clarifies how these groups differed. Measures to increase GPAPs participation should consider how ‘once at t1’ and ‘twice’ participation vary in terms of factors that influence or motivate participation. A small but growing proportion of the population participates in GPAPs. However, only certain population groups are reached by such programmes (e.g. females and those practising sports). Various steps are needed to achieve further effects and greater participation of groups at the population level that have not participated in GPAPs and are not ‘sportive’. It is not sufficient to simply expand programmes that have proven to be effective under research conditions [59]. As our study shows, it is important to tailor programmes to reach groups of both sexes and different levels of physical activity experience. In addition, programmes should be tailored to different groups in terms of their previous experience with GPAPs, and consider motivations and supportive/constraining conditions underlying participation in GPAPs and physical activity. This could be achieved by tailoring GPAPs to address potential participants’ readiness for change status or by considering the special needs of targeted groups or individual participants [9]. To get more people into GPAPs and physical activity, it seems promising to provide physical activity counselling for people with a poorer state of health and who present to primary care. There are positive reports from other countries [57] and the new Prevention Act introduced in 2015 in Germany promotes physician counselling [60]. Furthermore, our findings are also useful for other countries, as the non-communicable disease crisis is an overarching problem [1].

GPAPs should be tailored to build on previous experience of sports activities and to reach men as well as people with low education, groups that have been the least reached. GPAPs should also be complemented by policy and environmental interventions that support active living across society [9, 61, 62]. To upscale the effects and dissemination of GPAPs, it is necessary to address the contexts for healthy lifestyles with supportive health policies and enabling environments [59]. Furthermore, to achieve these goals it is necessary to reach the most disadvantaged populations through GPAPs and in related research [33]. Future research should concentrate on factors influencing longitudinal and long-term effects of GPAPs, information that is needed for research in promoting physical activity [63].

## Abbreviations

CI: Confidence interval
DEGS1: German Health Interview and Examination Survey for Adults 2008–11
GNHIES98: German National Health Interview and Examination Survey 1997–99
GPAPs: Group-based physical activity programmes
OR: Odds ratios
WHO: World Health Organization
